# Where does public funding for HIV prevention go to? The case of condoms versus microbicides and vaccines

**DOI:** 10.1186/1744-8603-6-23

**Published:** 2010-12-30

**Authors:** Anny JTP Peters, Maja Micevska Scharf, Francien TM van Driel, Willy HM Jansen

**Affiliations:** 1 Institute for Gender Studies, Radboud University Nijmegen, Netherlands; 2 Centre for International Development Issues Nijmegen, Radboud University Nijmegen, Netherlands; 3 Rutgers Nisso Group, Dutch Expert Centre on Sexuality, Utrecht, Netherlands; 4 Netherlands Interdisciplinary Demographic Institute (NIDI), The Hague, Netherlands

## Abstract

This study analyses the priorities of public donors in funding HIV prevention by either integrated condom programming or HIV preventive microbicides and vaccines in the period between 2000 and 2008. It further compares the public funding investments of the USA government and European governments, including the EU, as we expect the two groups to invest differently in HIV prevention options, because their policies on sexual and reproductive health and rights are different. We use two existing officially UN endorsed databases to compare the public donor funding streams for HIV prevention of these two distinct contributors. In the period 2000-2008, the relative share of public funding for integrated condom programming dropped significantly, while that for research on vaccines and microbicides increased. The European public donors gave a larger share to condom programming than the United States, but exhibited a similar downward trend in favour of funding research on vaccines and microbicides. Both public donor parties invested progressively more in research on vaccines and microbicides rather than addressing the shortage of condoms and improving access to integrated condom programming in developing countries.

## Background

The number of people living with HIV worldwide has continued to grow, reaching 33.4 million in 2008. In the same year 2.7 million new HIV infections occurred, almost half (45%) among people younger than 25 years [1]. Despite a more than eight-fold increase of total global financing for fighting AIDS, from 1.6 billion US$ in 2001 to 13.8 billion in 2008, a small fraction has gone to HIV prevention [2]. Public donor expenditures for treatment have grown much faster than the spending for prevention [3-5]. The two largest public AIDS funds, the Global Fund for HIV Tuberculosis and Malaria (GFATM initiated in 2001) and the Presidents' Emergency Programme for AIDS (PEPFAR since 2003), spend about 70% and 80% of their respective HIV budgets on treatment and care programmes in developing countries [6,7]. However, as of December 2008, mainly due to the high costs of treatment, 58% of those infected and requiring antiretroviral treatment cannot access such treatment [1]. Prevention, to halt the increase in new infections, therefore, remains as urgent as before. HIV experts currently agree that prevention is underfunded [3]. Therefore, insight into how the limited public funding for prevention is distributed is important.

At the end of 2008, for every two people starting antiretroviral treatment, five were newly infected [1]. Even if there were a cure for HIV, treatment only would by no means suffice to control the epidemic [8]. Although, HIV infection is avoidable, HIV prevention interventions are estimated to be accessible to fewer than one in five people worldwide [9]. Similarly, less than 40% of young people in developing countries are estimated to have basic information about AIDS and HIV prevention [1]. This knowledge gap might be due to the frequently expressed objections of political and religious leaders to sexual behavioural change programmes known to reduce HIV infection rates, such as integrated condom programming [10]. The same leaders, however, seem to be eager to welcome donor support to antiretroviral treatment for their populations [10]. The knowledge gap on prevention is reproduced on another level. Only a limited number of studies provide information on the coverage level of HIV prevention programmes in different developing countries [4], while ample data are available on the coverage rates of treatment and care programmes [1]. Public funders could play a crucial role in supporting developing countries to extend the coverage of evidence-based HIV prevention programmes. So might private and philanthropic donors, but due to lack of information on these funding streams they are excluded here from the analysis.

Within HIV prevention, different approaches can be distinguished, such as prevention by vaccines or microbicides, prevention by integrated condom programming, and some recently introduced prevention technologies such as male circumcision and prophylactic use of antiretroviral drugs. Prevention by vaccines or microbicides has been considered an important means to stop the AIDS epidemic since the beginning of the 21st century. Recently, the director of UNAIDS expressed his belief, that a preventive HIV vaccine holds the greatest opportunity for ending the epidemic and many share his view [11]. Several scientists, however, among them the chief editor of *the Lancet*, seriously question the possibility of developing a successful HIV preventive vaccine and criticise the overly optimistic prospect portrayed by the vaccine research community [12]. In 2007, five large-scale HIV vaccines studies were stopped because they failed to show satisfactory results [13]. In the same year, two microbicides trials were halted because they led to more HIV infections instead of less [14]. In 2009, vaccine researchers reported some success in a trial in Thailand, but the observed vaccine efficacy was too modest to be of any public health significance [15]. In 2010, microbicide researchers reported a first success in a trial in South Africa. Women who used the, to be tested microbicide were 39 percent less likely to become infected with HIV than women who received a placebo gel [16]. However, the consequences of these recent findings for prevention schemes are not clear yet and currently under discussion. Consequently, these technologies still are being researched and have not yet been applied in HIV prevention programmes. This means that the funding directed to this category of HIV preventives totally goes to research rather than to application in HIV prevention programmes, and therefore has not yet a direct effect on prevention.

Another HIV prevention technology is condoms and integrated condom programming. In contrast to vaccines and microbicides, male condoms have existed since at least 1000 BC. Female condoms, which are as effective as male condoms, have existed since 1984, and were officially approved by the United States Food and Drug Administration (US FDA) in 1993 [17,18]. In 2009, UNAIDS, WHO and UNFPA renewed their joint position statement on condoms: "The latex condom is the single, most efficient, available technology to reduce the sexual transmission of HIV" [19]. Empirically, its cost-effectiveness in comparison to other HIV prevention methods has been proven [20,21]. Female and male condoms are central to efforts to halt the spread of HIV. This was officially recognized as early as 1994 in the Programme of Action of the International Conference on Population and Development [22]; again in 2001 in the Political Declaration of commitment on HIV/AIDS in the United Nations General Assembly Special Session (UNGASS) on HIV/AIDS [23]; and again in 2005 as part of a plan to achieve the Millennium Development Goals [24]. The female condom in particular is currently the only technology that gives women greater control over protecting themselves from HIV, other STIs and unintended pregnancy [25,26].

Integrated condom programming is essential to the realisation of sexual and reproductive health and rights, including the prevention of HIV [27-29]. Integrated means that the programme is delivering two or more types of services previously provided separately, as a single, coordinated, and combined service. Examples are condom programming combined with counselling services on family planning, or with HIV/STI testing services or with sexuality education [30]. Integrated condom programming has proven to be successful, under the condition that a gender, relational and community perspective is used [31,32]. And that the condoms are affordable. Cost studies have shown that the consumer price of condoms has a strong effect on access and, thus, usage [33]. Integrated programmes, which subsidise or freely distribute condoms, have led to increased usage, a condition for effectiveness in HIV prevention [34]. In July 2010, during the last international AIDS conference in Vienna, UNAIDS reported on successes in HIV prevention by integrated condom programming in a multi country study [35]. At the same conference, a researcher from John Hopkins University showed convincing results of declining HIV infection rates in countries with generalised HIV epidemics. These declines occurred in a time when antiretroviral treatments were not yet available and when priority was on prevention through sexual behavioural change programmes combined with unproblematic access to condoms [36]. It is beyond the scope of this article to discuss the factors leading to successful condom programming in-depth. However, it is important to recognise that it is an evidence-based, cost-effective, efficient, and directly available way of delivering HIV prevention services to people.

Apart from the two above mentioned prevention approaches, three other HIV prevention technologies were introduced in some developing countries such as male circumcision [37], use of anti-retroviral drugs in pregnancy to prevent mother to child transmission (PMCT) [38], or prophylactic use of antiretroviral drugs (PreP) [39]. All three technologies will only be partly efficacious for preventing sexual transmission of HIV. Circumcised men may still contract HIV (and other STI's), and can still pass it on to their next partner, making protection with condoms still necessary and thus, the need for integrated condom programming. The need for protection also remains when introducing PMCT or PreP. There is still much discussion on the assessment of the effectiveness of the various HIV prevention technologies. The assessment varies with the researchers' disciplinary perspective. Kippax concluded in her study that the (bio-) medical sciences are dominant in the discussion on HIV prevention, leaving hardly any space for social sciences [40]. In this article, we will not address the different scientific interpretations in HIV prevention effectiveness, since this is done recently by Heise et al [41]. We will focus on the funding choices being made in HIV prevention. Our first question is therefore: How is the public funding from USA and Europe for HIV prevention divided over research on HIV preventive vaccines and microbicides and integrated condom programming in the period 2000 to 2008?

We are particularly interested in the funding choices in HIV prevention, taken by two different public donors, the USA government, and European governments, including the EU. The European public donors have a long tradition of supporting gender and sexual and reproductive health and rights as part of Official Development Aid [42,43]. The following statement on HIV prevention of the Council of the European Union illustrates its position:

We re-affirm our commitment to tackle the HIV pandemic in a comprehensive and integrated way and in particular the HIV prevention gap. We are profoundly concerned about the resurgence of partial or incomplete messages on HIV prevention, which are not grounded in evidence and have limited effectiveness. We, the European Union, firmly believe that HIV prevention must utilise all approaches known to be effective, like universal access to sexual and reproductive health information in accordance with the international decisions at the International Conference on Population and Development agenda and reliable access to essential sexual and reproductive health commodities, including male and female condoms [44].

The European donors thus clearly recognise the importance of sexual and reproductive health and rights, and explicitly state the necessity to provide reliable access to male and female condoms. In contrast, the USA government failed to set up a holistic sexual and reproductive health and rights approach in development aid. In the period between 2000 and 2008, especially with the Bush presidency, it has implemented a conservative HIV policy leading to a global anti-condom movement [45,46], started earlier by the Catholic Church. Because of this difference in policy, we expect to find that the European governments and the EU give a larger share of the funding to integrated condom programming in the period 2000 - 2008 than the USA. Our second question therefore is: Is there a difference in public donor funding within HIV prevention between the EU and the USA?

## Methods

We compare the actual amounts and relative share of public funding by the USA and Europe for two categories of HIV prevention. This comparison limits itself to public funds donated to HIV prevention by donor governments, i.e. public donors. Private and philanthropic donors are not included in our study for several reasons. Firstly, the public donors have a responsibility to take measures for HIV prevention and for the development of HIV prevention technologies. New HIV technologies are mainly being developed with public sector financing and not private sector funding [41]. Secondly, public donors are primarily accountable in relation to the effectiveness and efficiency in HIV prevention, especially under Official Development Aid (ODA). Data availability is a third reason. While data on public funding are relatively easily detectable, data on private funds are scattered and no integrated database exists containing all the foundations active in the field. Public donors are primary donors. These primary donors provide the basic information for our study. We review actual donor government expenditures in support of HIV prevention for two groups of primary donors, i.e. European governments including the European Commission, and the USA. The funding comes directly from these public funding agencies, and is directed to research bodies and international development assistance agencies.

This study is based on secondary analysis, using information from two available databases, of which UNAIDS endorses one and UNFPA the other [47,48]. We did not gather new data, but categorise, compare and analyse existing data.

Tracking donor government financing for HIV vaccines and microbicides is relatively easy since these two prevention methods are still in the stage of research and not in delivery and, thus, are not yet part of daily programming. It is relatively easy to classify financial support to research and trials, which have a clear start and ending. Computing donor government funding levels for integrated condom programming is more complicated, because of its integration in different programmes and services. Condoms offer dual protection: against unwanted pregnancy and against sexually transmitted infections. Consequently, integrated condom programming is an essential component of family planning, reproductive health, and AIDS interventions. In our study, we do not distinguish financial flows for integrated condom programming used for family planning and reproductive health purposes from those used for HIV prevention, as we will elaborate below.

The data on funding for research on HIV vaccines and microbicides are collected on an annual basis by the HIV Vaccine Microbicide Resource Tracking Group, which consists of three organisations: the HIV Vaccine Advocacy Coalition (AVAC), the Alliance for Microbicide Development (AMD), and the International HIV Vaccine Initiative (IAVI) supported by UNAIDS [47]. To analyse the financial resource flows for integrated condom programming, we used the database of the UNFPA/NIDI project "Financial Resource Flows for Population and HIV activities" as a source http://www.resourceflows.org. This project monitors the global financial flows allocated to sexual and reproductive health and rights, including AIDS, to assess the fulfilment of commitments made at the International Conference on Population and Development (ICPD) Programme of Action, in 1994, and at the UNGASS on HIV/AIDS in 2001, as described earlier. On an annual basis, UNFPA/NIDI report and present their data to the UN Secretary-General [48]. The UNFPA/NIDI database, like the one from the HIV Vaccine Microbicide Resource Tracking Group, tracks financial resource flows of primary donors, among others. Their data are comparable because they both use the same definition and categorisation of donors. Moreover, both databases make use of the same research methodology, surveying donors by using self-administered standard questionnaires about their funding streams. However, calculating the exact funding for condom programming from the UNFPA/NIDI database was not self-evident, because condoms are often an integrated part of a project and thus calculation of funding levels for integrated condom programming requires certain estimates. We used the following approach to reach the best estimates.

In the UNFPA/NIDI questionnaire, donors categorise their funding in line with that of the 1994 and subsequent ICPD programmes of action as follows: family planning, AIDS, reproductive health, and basic research. Integrated condom programming can be part of any of these four categories. By far the majority of projects are classified as mixtures, meaning that they fall in two or more of the four categories, expressed in percentages. Thus, we considered all four categories equally to find the total funding for integrated condom programming. The UNFPA/NIDI database contained 6,707 projects in 2000, which increased to 15,098 projects in 2008 (Table 1. Column A). The total amount of funding increased from 1,887 million US$ in 2000 to 10,778 million in 2008 (Table 1. Column B). To establish the integrated condom projects, we counted the number of projects with the word "condom(s)" and "contraceptive(s)" in the project title and/or in the project description, which typically summarizes the project in about 300 words. We assumed that if there is no mention of "condom(s)" or "contraceptive(s)" in the title or description of a particular project, condom programming is not part of the project. This resulted in 294 projects in 2000 and 68 in 2008 (Table 1. Column C), and total amounts of funding of 189 m US$ and 42 m US$, respectively (Table 1. Column D). Among the total number of projects, there were many without or with a very short project description of less than 50 characters, and thus with a little chance of including the words "condom(s)" or "contraceptive(s)". We therefore discarded all these projects and only took into account the projects with a full project description of more than 50 characters, which had a sufficient chance to contain the words "condom(s)" or "contraceptive(s)" and describe the integration of condoms in the project (Table 1. Column E). For each year, we calculated the proportion of the projects with either one of these words by dividing column C (number of projects with "condom(s)" or "contraceptive(s)" in the title or description) by column E (total number of projects with a project description of more than 50 characters) and multiplied by 100 to find the percentages (Table 1. Column F). We multiplied this percentage with the total amount of public donor expenditure on family planning, AIDS, reproductive health and basic research (Column B), assuming that the projects without any project description, or a very short one, were similar to the projects with descriptions.

**Table 1:** Estimating public donor expenditures for integrated condom programming 2000 - 2008 using UNFPA/NIDI database (million US$).

	A	B	C	D	E	F	G
Year	Total # of projects	Total amount (m US$)	# Projects with "condom(s)" or "contraceptives" in project title/description	Total funding of projects with "condom(s)" or "contraceptives" in title/description (m US$)	# Projects with project description of >50 characters	% Projects with "condom(s)" or "contraceptives" in title/description	Estimated amount spent on integrated condom programming (m US$)
2000	6,707	1,887	294	189	2,456	12.0	226
2001	7,421	2,103	153	135	2,388	6.4	135
2002	8,610	3,225	168	129	2,911	5.8	186
2003	11,079	3,845	116	59	1,503	7.7	297
2004	8,981	4,813	81	65	2,865	2.8	136
2005	11,576	6,891	133	123	2,885	4.6	318
2006	18,522	7,381	93	110	5,714	1.6	120
2007	13,860	8,806	25	83	8,904	0.3	25
2008	15,098	10,778	68	42	7,936	0.9	92

The figures presented in Column G are currently the best available estimates for the total public donor expenditures for integrated condom programming. We use these figures to compare the funding streams of the EU and USA on integrated condom programming. Still, one should be aware of the assumptions made in calculating these figures and consider that we are interested in the observed trends, rather that the precise data for a particular year.

Since we estimated the volume of integrated condom programming, we considered it important to add sources for counterchecking these estimates. Additionally, we studied the global trends in donor purchases of condoms in the period 2000 - 2008 by using the annually produced UNFPA reports called "Donor support for contraceptives and condoms for STI/HIV prevention" [25]. This report is based on a database produced by the commodity management branch of UNFPA, which directly collects data from donors on the procurement and international transport of condoms. http://rhi.rhsupplies.org. However, this database is not suitable to compare data between the USA and Europe, because it does not make a clear distinction between primary and secondary donors. This latter group includes for example international NGOs, whose funding originates from primary donors, making original funding from USA or European governments indistinguishable. We also compare our results with the estimated shortages of condoms in developing countries, as described in literature.

## Results

**Figure 1:**
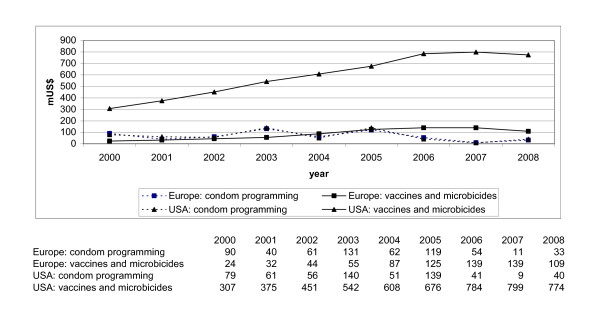
Trends in donor expenditures for vaccines and microbicides vs. for integrated condom programming from Europe and the USA 2000 - 2008 (m US$).

Figure 1 shows the amounts and trends in donor government financing for our two categories of HIV prevention: 'vaccines and microbicides' and 'integrated condom programming' by primary donors: the governments of Europe, including the EU, and the government of the USA.

Both Europe and USA increased funding to research into vaccines and microbicides between 2000 and 2008. The USA has constantly and steeply increased their funding to research into vaccines and microbicides, from 307 m US$ in 2000 to 799 m US$ in 2007, and a slight decrease in 2008 to 774 m US$. A constant rise in funding for this sector is also evident for Europe: from 24 m US$ in 2000 to 139 m US$ in 2007, and a slight decrease in 2008 to 109 m US$. Moreover, USA funding to research in vaccines and microbicides is significantly higher than European funding. Both Europe and USA decreased funding to integrated condom programming in the period 2000-2008, in a similar way and Figure 1 does not show any difference in these two trend lines. They are rather overlapping. USA and Europe gave about the same amount of funds to integrated condom programming, although irregular. The USA decreased their funding between 2000 and 2008 from 79 m to 40 m US$. In 2008, funding was about 50% under the level of 2000. European governments decreased their funding to the delivery of integrated condom programming from 90 m US$ in 2000 to 33 m US$ in 2008 and, like the USA, in 2008 ended under the level of 2000. Figure 1 also shows that financing priorities of governments in Europe have shifted from integrated condom programming to research into vaccines and microbicides between 2003 and 2004. It also shows that 2008 might be the beginning of a shift towards slightly increased investments in integrated condom programming.

For a further interpretation of the quantity of public donor expenditures by Europe and the USA, it is important to consider the size of the respective populations and economies. Between 2000 and 2008, the countries that had the largest contributions to the total of sexual and reproductive health and rights including AIDS were the United Kingdom, the Netherlands, Norway, Denmark, Finland, and Sweden, each contributing between 400 and 800 US$ per million dollars of gross national income (GNI) [49]. Within Europe, there are also differences. Norway contributes almost four times as much as Italy, despite having a six times smaller economy. The Netherlands contributes more than six and half times as much as France, although its economy is less than a third of that of France [42]. The American government gave about half of the average amount of European countries: between 200 and 400 US$ per million dollars of GNI [49].

**Figure 2:**
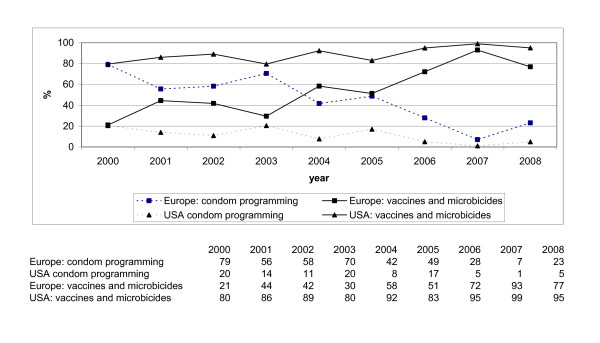
Relative share of funding for vaccines and microbicides vs. for integrated condom programming by governments from Europe and USA 2000 - 2008 (%).

Figure 2 shows that, Europe shows a similar decrease in the share of funding to implementation of integrated condom programming as the USA: the share of funding to integrated condom programming by Europe decreased from 79% to 23% between 2000 and 2008. The share of funding to integrated condom programming by the USA decreased, with fluctuations, from 20% to 5%. Our results show a turning point in 2004 in funding practices for the European governments: before 2004, the majority of funding goes to integrated condom programming while after 2004 research into vaccines and microbicides increasingly receives more. It is noteworthy that Europe shows a similar sharp reduction of their financial support of condom programming as the USA and its conservative condom sentiments.

**Figure 3:**
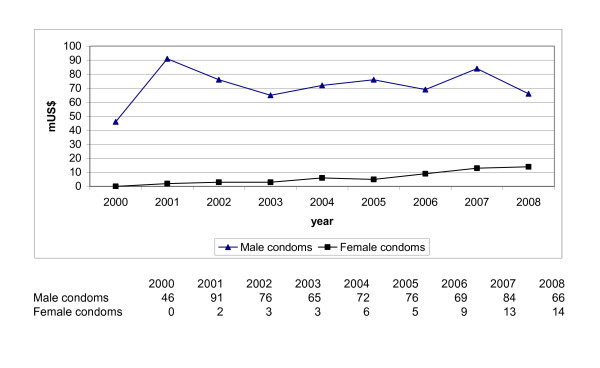
Trends in total donor expenditures for male and female condoms 2000 - 2008 based on UNFPA database (m US$).

Our countercheck, described in our methods paragraph reveals the global trends in total donor funding for male and female condoms in the period 2000 - 2008, as shown in Figure 3.

Donor expenditure on male condoms is relatively constant over these nine years, on female condoms increasing. Our observed trend in decreased funding for integrated condom programming is not contradictory to the trend in total donor funding for only the purchase of male and female condoms. Most years the funding spent on integrated condom programming is 2 to 4 times more than the money spent on the purchase of condoms. This means that programming costs are 2 to 4 times the costs of buying the commodities. Our results also match a same type of trend in global condom shortfalls as analysed in a few other studies [25,50]. A global condom shortage existed before 2000 and sustained during the period under research. UNFPA calculated a shortfall of 7 billion male condoms in developing countries in 2000 [51] increasing to 16 billion in 2006, mainly due to increased population figures [52]. The global shortfall of condoms exists despite an increased provision of condoms by the private sector. Middle income countries such as Brazil, China, India, and South Africa do not depend on foreign public donors for their condom supply, unlike some low income developing countries [53-55]. We did not observe a significant increase in public donor support for integrated condom programming in relation to this existing and increasing condom shortfall. The current shortfall of the female condom is much higher than of the male condom [26]. Above data shows some increased funding for female condoms, but this amount remains minimal in relation to the rest of the amounts.

## Discussion

Our study leads to a new insight in the trends in public funding on HIV prevention. There is a remarkable shift away from supporting low cost and effective technologies to funding the research into as of yet not proven high technology HIV preventives. Moreover, our expectation that the European donors let themselves be guided by their sexual and reproductive health and rights policies and their claims for universal access to condoms, proved incorrect. Unexpectedly, they have decreased their relative share of funding to condom programming in times that the AIDS problem exploded further. It looks as if the European public donors now follow the American prevention agenda and move away from the 1994 programme of action of the ICPD, specifically from its integrated condom programming [56,57].

Although, we are not in a position to fully discuss the determining factors behind the found public funding trends on HIV prevention, we like to consider a few. This enables the readers to place our findings in a broader context. One such factor might originate from the 2001 United Nations General Assembly Special Session (UNGASS) on HIV/AIDS. This assembly ended with a declaration of commitment on HIV/AIDS [23]. Afterwards, UNAIDS developed indicators, aimed to monitor global progress on this declaration of which only one is related to HIV prevention: "the level of public sector investment in research and development (R&D) for HIV vaccines and microbicides" [58]. Reference to fund other HIV prevention strategies, such as integrated condom programming is absent. We assume that this global indicator made American and European donors alike raise investments in research into vaccines and microbicides.

The position taken by UNAIDS, the global lead agency on AIDS, might also contribute to diminishing investment in integrated condom programming by the European donors. Although UNAIDS mentions condom promotion in its HIV prevention policy, it does not prioritise or highlight integrated condom programming [56]. Even the most recently published UN progress report "Scaling up priority HIV/AIDS interventions in the health sector" totally ignores integrated condom programming [57]. Indeed other researchers earlier pointed to the weak promotion of condoms by UNAIDS. They literally speak about "the virtual disappearance of condom promotion in UNAIDS literature and campaigns" [4].

Another factor, mentioned in recent studies [59], is the influence of philanthropic donor organisations on public donors. Specifically, the priorities of the biggest private AIDS donor organisation in the USA, the Bill and Melinda Gates Foundation (BMGF), might have an impact on government funding policies. Globally, USA government and BMGF account for 79% of the global funding for vaccines and for 59% of the global funding for microbicides [47]. The two agencies have a same type of HIV prevention funding focus, namely new, biomedical technologies, such as vaccines and microbicides [59].

Our findings clearly demonstrate a global under-exploitation of integrated condom programming, a phenomenon heavily debated in the context of global health governance [60,61]. Some scholars link such priority shift in funding HIV prevention to economic and scientific interests of the donors [62,63]. Recipients of funding for integrated condom programming are above all the governments of developing countries or NGOs [48]. Recipients of funding for vaccines and microbicides, are primarily privately owned medical pharmaceutical companies or scientific research institutes based in North America and Western Europe, with associations in developing countries [62,63]. Illustrative is also the title of the new annual report of HIV Vaccines and Microbicides Resource Tracking Working Group: "Advancing the Science in a Time of Fiscal Constraint: Funding for HIV Prevention Technologies in 2009" [47]. The advancement of science clearly is different from the advancement of HIV prevention in the context of development assistance. In terms of official development assistance, concern is expected to be with women and men in developing countries who daily run the risk of infection and urgently need access to low cost and effective HIV preventive means and programming. They should not be left in the cold with only the promise of a forthcoming 'biomedical magic bullet to solve HIV'. Other scholars have noted a bias in favour of biomedical research rather than an investment into socio-cultural studies that re-examine sexuality and gender relations to better implementation of condom programming [40,62]. Further research into the power and gender issues that are at play in the decision-making on public funding for HIV prevention is necessary.

## Conclusion

The governments of the USA and Europe (European countries and the EU) both shifted their attention from funding of integrated condom programming to research into new prevention technologies, such as vaccines and microbicides. We revealed a disturbing unexpected trend in funding from the group of European public donors in contrast with their fierce fight for the ICPD programme of action of 1994. The tendency that American and European donors are both increasingly reluctant to commit sufficient funds for sexual and reproductive health and rights has been concluded earlier [63]. Our study adds the revealing conclusion that the European donors have relatively cut funding on integrated condom programming to the same extent as the USA.

## Recommendations

We recommend that public funders aim at a clear insight in the funding trends and reflect on the consequences of the shifts in these trends and what they actually mean for the people in need for HIV prevention.

We recommend that increasing funds for developing one type of HIV preventive should not be detrimental to the support for another, an already effective means of protection, as long as these are not yet generally available and accessible. Public funders should better realise that education and access to condoms remain a central priority issue in HIV prevention.

We recommend that public funders who like to adhere to sexual and reproductive health and rights policies not only monitor and extend funding for integrated condom programming, but also show the value of sociological research for the successful implementation of HIV prevention and integrated condom programming.

Further research is necessary to understand better why public donors make certain funding choices on HIV prevention for developing countries, and particularly to assess how power and gender issues are involved in decision making on funding for HIV prevention.

## Competing interests

The authors declare that they have no competing interests.

## Authors' contributions

AJTPP coordinated and conducted the study and drafted the manuscript.

MMS performed the tailor-made data-analysis of the UNFPA/NIDI project "Financial Resource Flows for Population and HIV activities" and participated in the design of the study.

FTMVD and WHMJ participated in the design of the study and commented on the manuscript.

All authors read and approved the final manuscript.
